# Regulatory Response to Carbon Starvation in *Caulobacter crescentus*

**DOI:** 10.1371/journal.pone.0018179

**Published:** 2011-04-11

**Authors:** Leticia Britos, Eduardo Abeliuk, Thomas Taverner, Mary Lipton, Harley McAdams, Lucy Shapiro

**Affiliations:** 1 Department of Developmental Biology, Stanford University School of Medicine, Stanford, California, United States of America; 2 Department of Electrical Engineering, Stanford University, Stanford, California, United States of America; 3 Environmental Molecular Sciences Laboratory, Pacific Northwest National Laboratory, Richland, Washington, United States of America; East Carolina University School of Medicine, United States of America

## Abstract

Bacteria adapt to shifts from rapid to slow growth, and have developed strategies for long-term survival during prolonged starvation and stress conditions. We report the regulatory response of *C. crescentus* to carbon starvation, based on combined high-throughput proteome and transcriptome analyses. Our results identify cell cycle changes in gene expression in response to carbon starvation that involve the prominent role of the FixK FNR/CAP family transcription factor and the CtrA cell cycle regulator. Notably, the SigT ECF sigma factor mediates the carbon starvation-induced degradation of CtrA, while activating a core set of general starvation-stress genes that respond to carbon starvation, osmotic stress, and exposure to heavy metals. Comparison of the response of swarmer cells and stalked cells to carbon starvation revealed four groups of genes that exhibit different expression profiles. Also, cell pole morphogenesis and initiation of chromosome replication normally occurring at the swarmer-to-stalked cell transition are uncoupled in carbon-starved cells.

## Introduction

Starvation for nutrient and energy sources are common stresses confronted by bacteria in natural environments. Bacteria have limited energy reserves, so they need robust mechanisms to quickly shift between rapid and slow growth, as well as a strategy for long-term survival during periods of prolonged starvation. The response to starvation comprises an initial stage of scavenging and metabolic adaptation. If the missing essential nutrients are not replenished, there is a second stage of physiological adaptation, which includes the inhibition of growth and cell division, in order to retain viability.

We have used *Caulobacter crescentus*, a gram-negative oligotrophic bacterium [1], as a model system to study the response to carbon deprivation. *C. crescentus* has a dimorphic life cycle. Each asymmetric division yields a chemotactically-competent flagellated cell (swarmer cell) and a sessile cell with a polar stalk (stalked cell). The core genetic network that drives cell cycle progression and cell division in *C. crescentus* is well characterized [2], [3], [4], [5].

In *Escherichia coli* and *Bacillus subtilis*, the RpoS (σ^S^) and SigB (σ^B^) sigma factors are the master regulators of the general starvation-stress response [6], [7]. *C. crescentus* lacks orthologs of the *rpoS* and *sigB* genes, as do all other α-proteobacteria [8], [9]. An equivalent master regulator has not been identified. We have recently identified two factors involved in the adaptation of *C. crescentus* to carbon starvation: the SpoT ppGpp synthetase/hydrolase, which contributes to the regulation of the DnaA protein stability and initiation of DNA replication [10], and the CrfA small noncoding regulatory RNA controlling the mRNA stability of 27 transcripts [11].

A comparison of gene expression profiles of *C. crescentus* growing under carbon- and nitrogen-limited continuous flow cultures was recently reported [12]. In these experiments, slow cell growth was supported by a constant supply of nutrients at low concentration, and the gene expression profiling captured the metabolic adaptations to the relative levels of carbon and nitrogen. This study identified genes differentially induced in carbon- versus nitrogen-limited conditions, among which are those predicted to require the alternative sigma factor RpoN for complete induction [12]. Here, we have studied the cells' response to an abrupt loss of carbon source resulting in the inhibition of mass accumulation and cell cycle progression. We performed a global analysis of the differences between growing and carbon-starved *C. crescentus* cultures, through combined high-throughput proteome and transcriptome assays. By analyzing the response at the protein level, we took into account the fact that *C. crescentus* uses a complex array of regulatory strategies that include targeted proteolytic events [13]. We identified genetic regulatory pathways that mediate the transduction of carbon starvation signals and found that the SigT ECF sigma factor controls a core set of genes that are activated by carbon depletion, osmotic stress and exposure to heavy metals. We identified gene clusters that are differentially expressed upon carbon starvation at specific stages of the cell cycle.

## Results

### The proteome and transcriptome profile of *C. crescentus* cells starved for carbon

Transfer of *C. crescentus* cultures in exponential growth to media lacking a carbon source results in immediate growth arrest. Under prolonged starvation, there is a pronounced loss in viability [10]. In order to identify proteins that participate in the carbon starvation response, we incubated cultures in minimal M2 medium in the absence of glucose, and the respective non-starved controls in the presence of 0.2% glucose, for 30 and 60 minutes, as described in the Methods section. Samples were analyzed by liquid chromatography coupled to tandem mass spectrometry. We identified 2471 distinct proteins across all conditions tested, accounting for 65.6% of 3767 *C. crescentus* predicted protein-coding genes. This is high coverage for a prokaryotic proteome, lower only than that reported for *Mycoplasma mobile* (88.6%), which has a genome of one-sixth the size of that of *C. crescentus* (approx 0.78 Mb) (Table S1). Previous electrophoresis-based proteome studies of *C. crescentus* identified 81 cell cycle-regulated proteins [14], 39 stalk-specific proteins [15], and 86 membrane-associated proteins [16], [17].

**Figure 1:**
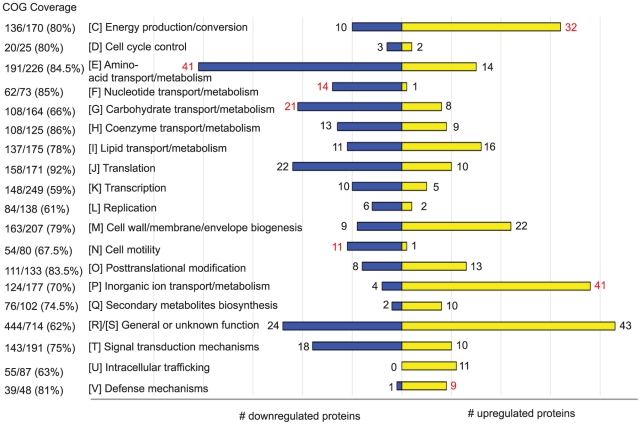
Proteome profile of *C. crescentus* subjected to carbon starvation. Proteins that change significantly upon 30 and/or 60 minutes of carbon starvation classified by NCBI Clusters of Orthologous Genes (COG). Coverage for all categories of the COG scheme (except for A and B for which no proteins were detected), is indicated as the ratio of the number of detected proteins in each category over the total number of proteins assigned to that category (the corresponding percentage value is indicated in parentheses). Yellow bars represent upregulated proteins and blue bars, downregulated proteins in each COG category. Values that meet a statistically significant enrichment, assuming a hypergeometric distribution, are denoted in red. The proteins within each category are listed in Table S2, along with 43 additional proteins that are not included in the COG classification.

The levels of 513 proteins were found to change reproducibly in cells starved for carbon for 30 and/or 60 min (Table S2). Figure 1 shows the upregulated and downregulated proteins by functional categories, according to COG classifications [18]. Those categories with a statistically significant enrichment in either upregulated or downregulated proteins are indicated (see Methods for details on the enrichment analysis). Functional categories comprising transport and metabolism of amino acids (COG E), nucleotides (COG F), carbohydrates (COG G) and cell motility (COG N) were significantly enriched in proteins whose levels decreased upon carbon starvation. On the other hand, the categories comprising energy production and conversion (COG C), inorganic ion transport and metabolism (COG P) and defense mechanisms (COG V) were significantly enriched in proteins whose levels increased upon carbon starvation. Of the 40 COG P proteins that are upregulated upon carbon starvation, 21 belong to the TonB-dependent receptor family, which are highly abundant in the *C. crescentus* genome, including those controlled by the CrfA small noncoding RNA described by Landt *et. al.* [11]. These observations are compatible with metabolic adaptation of the cell to carbon starvation.

The proteome profile of carbon-starved cells also provided insights into the physiological adaptation associated with stasis. Among the proteins that change significantly, there was notable downregulation of three essential cell cycle regulators (CtrA, DnaA and GcrA), and two proteins (PopZ and ParA) that mediate chromosome segregation [13], [19], [20]. The decrease in the levels of DnaA, an essential protein that serves both as an activator of replication initiation and global transcription factor [21], is consistent with the previously reported starvation-induced ClpP-mediated proteolysis of DnaA [22]. The decrease in DnaA levels prevents the initiation of DNA replication. The CtrA response regulator inhibits DNA replication in the swarmer cell, and acts as a transcription factor regulating the expression of nearly 100 genes. It was previously shown that CtrA is proteolyzed in swarmer cells starved for carbon, but with different kinetics than that observed under normal growth conditions [10], [22]. Given the number of CtrA-regulated genes that change upon carbon starvation, it is clear that, at least in part, an important role of CtrA in the starvation response relies on its function as transcription factor. Additionally, the level of the FtsZ cell division protein, that is essential for cytokinesis, is shown to decrease significantly upon carbon starvation; FtsZ was previously shown to decrease during stationary phase in *C. crescentus* [23].

Total RNA was extracted from samples parallel to those used for the proteome analysis. As described in Methods, cDNA was synthesized, fragmented, labeled and hybridized to the CauloHI1 chip. A minimum two-fold change requirement between the starvation and non-starvation conditions, with a false discovery rate (FDR) below 1%, resulted in upregulation of 607 of the 3767 *C. crescentus* protein coding genes after 30 min of carbon starvation, and 700 genes after 60 min (553 of these were significantly upregulated at both time points); 725 and 618 genes were downregulated after 30 and 60 min of carbon starvation, respectively; 603 genes were equally downregulated for both time points. All genes that changed significantly are listed in Table S3. 16.3% of the 753 genes that were significantly upregulated after either 30 or 60 min of carbon starvation, and 19.9% of the 739 downregulated genes were classified as cell cycle regulated transcripts by Laub *et al.* [24] (Table S3).

### Carbon starvation transcriptional regulators

**Figure 2:**
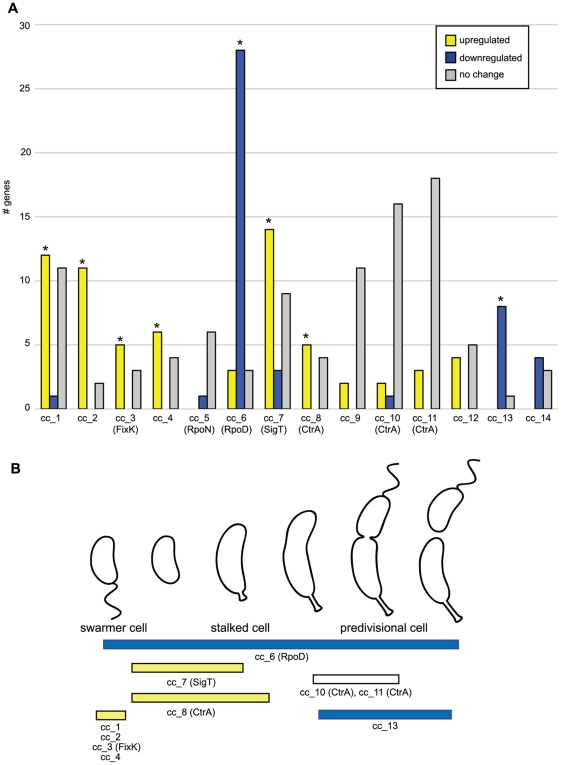
Putative regulators of the carbon starvation response. **A**. Previously identified clusters of *C. crescentus* genes that share conserved promoter motifs were analyzed for enrichment in genes that change significantly upon carbon starvation. For each gene set, cc_1 through cc_14 (identified by a shared promoter motif), the number of carbon starvation upregulated genes is indicated in yellow, downregulated genes in blue and genes that don't change in gray. Asterisks denote statistically significant enrichment. Gene numbers correspond to 30 min of carbon starvation, except for motif cc_7, for which gene numbers correspond to 60 min of carbon starvation. Identical results were obtained in terms of significant enrichment for the 30 and 60 min carbon starvation data for most gene sets, except for cc_8, which was only enriched in starvation upregulated genes at 30 min, and cc_7, which was only enriched at 60 min. **B**. Cell cycle patterns of expression, as previously determined [25], of carbon-starvation enriched gene sets. Yellow indicates enrichment in genes up-regulated upon 30 min of carbon starvation, while blue corresponds to enrichment in genes down-regulated upon 30 min of carbon starvation.

Cell cycle transcription profiles were previously used to identify predicted cell cycle regulons and their conserved promoter motifs. Fourteen conserved promoter motifs were identified, seven of which shared significant similarity with binding motifs for previously characterized regulators [25]. We analyzed these sets of genes to determine if any of them were significantly enriched in genes whose expression was up- or down-regulated upon carbon starvation. As shown in Figure 2A, out of 14 gene sets, each of which shared a distinct promoter motif, six were significantly enriched in genes upregulated upon carbon starvation (sets corresponding to motifs cc_1, cc_2, cc_3, cc_4, cc_7, cc_8), while two were enriched in genes downregulated upon carbon starvation (corresponding to motifs cc_6 and cc_13) (Table S4). Motif cc_6 is the cognate binding motif for the RpoD sigma factor, which drives the expression of biosynthetic and housekeeping genes throughout the cell cycle [26] (Figure 2B); as expected, the great majority of the genes with cc_6 motif (27 out of 34) were down-regulated upon carbon starvation in our experiments.

#### Carbon starvation FixK regulon

Genes with motifs cc_1 to cc_4 are expressed in the swarmer cell stage (Figure 2B). A subset of these genes are likely activated by the stress suffered by cells during the synchronization process, and not swarmer cell specific genes, as they were activated equally by carbon starvation in stalked cells. Motif cc_3 corresponds to the DNA binding motif of the FixK transcription factor. Microarray analysis of a *C. crescentus fixK* knockout strain identified downstream targets whose expression changed in a FixK-dependent manner under hypoxia conditions [27]. We found that five out of eight genes with motif cc_3 were upregulated upon 30 min of carbon starvation. Expression of three of those genes was FixK-dependent under hypoxia conditions [27]. Eleven additional FixK-dependent genes identified by Crosson *et al.*, which lack the cc_3 motif, showed a significant change upon carbon starvation, and are either indirect FixK targets, or are transcribed as part of an operon directly regulated by FixK.

#### Carbon starvation SigT regulon

Motif cc_7 is the binding motif for the SigT alternative sigma factor, and genes in this cluster have a distinct peak of expression at the swarmer-to-stalked cell transition (Figure 2B). SigT, which belongs to the ECF (extra-cytoplasmic function) sigma factor family, is a regulator of the osmotic and oxidative stress responses in *C. crescentus* [28]. Of 26 genes with motif cc_7, 7 were upregulated after 30 min of carbon starvation, and an additional 7 genes were upregulated after 60 min.

In order to characterize the carbon starvation SigT regulon, we performed microarray experiments comparing gene expression profiles of a *C. crescentus* wild-type strain (LS101) and a *sigT* null mutant strain (LS3554), in M2 cultures incubated in the absence of glucose for 15 min. Genes showing a greater than two-fold difference in expression between the mutant and the wild type strain under starvation conditions, and meeting statistical significance as defined in the Methods section, were considered to be SigT-dependent upon carbon starvation (Table 1). Thirteen of the 27 SigT-dependent carbon starvation genes have the previously characterized [25], [28] SigT binding motif in their promoters and are presumably direct targets of the regulator. Four additional targets are in close proximity to genes that possess the motif, suggesting they are part of directly regulated operons. Sixteen of the 27 genes whose expression is significantly reduced in the *sigT* mutant, and the two genes with increased expression in the mutant strain, do not show SigT-dependency under osmotic stress, as reported by Alvarez-Martinez *et al.* [28].

**Table 1:** SigT-dependent carbon starvation regulon.

Gene	Annotation	COG	Fold reduction in Δ*sigT* mutant	SigT motif?	Sig-T dependent in osmotic stress?	Up-regulated by exposure to multiple heavy metals?
CC_0284	Two-component receiver domain protein lovR	K	2.65	Yes	Yes	No
CC_1356	Transcriptional regulator	K	3.29	Yes	Yes	No
CC_2883	RNA polymerase ECF-type sigma factor sigU	K	15.93	Yes	Yes	Yes
CC_3477	Hybrid sigma factor/two-component receiver protein phyR	K	3.10	Yes	No	No
CC_0285	Photosensory histidine protein kinase lovK	T	3.94	No (*)	No	No
CC_2330	HTH transcriptional regulator	K	2.25	No	No	No
CC_3258	chemotaxis receiver domain protein cheYIII	K	2.05	No	No	Yes
CC_2549	Putative heme-binding protein	NC	3.97	Yes	Yes	Yes
CC_1048	Acylamino-acid-releasing enzyme	E	0.42	No	No	No
CC_2318	ABC transporter permease protein	Q	2.15	No	No	No
CC_3001	TonB-dependent receptor	P	2.08	No	No	No
CC_3572	Carbonic anhydrase	P	0.43	No	No	No
CC_0280	Conserved cell surface protein	NC	20.99	Yes	Yes	Yes
CC_0673	Hypothetical protein	NC	3.59	Yes	No	Yes
CC_0717	Hypothetical membrane associated protein	S	2.01	Yes	No	No
CC_1179	hypothetical protein	NC	3.14	Yes	Yes	Yes
CC_0163	Conserved hypothetical protein	S	5.60	No (*)	Yes	No
CC_1178	Hypothetical cytosolic protein	R	2.10	No (*)	Yes	Yes
CC_0557	Hypothetical protein	NC	2.78	No	No	No
CC_1532	Conserved hypothetical protein	S	23.41	No	Yes	Yes
CC_3466	Hypothetical protein	NC	7.67	No	No	Yes
CC_3473	Entericidin B homolog	NC	2.57	Yes	No	Yes
CC_1682	Transglycosylase associated protein	S	2.57	Yes	No	Yes
CC_0201	Outer membrane protein	M	2.11	Yes	Yes	Yes
CC_0428	Methyl-accepting chemotaxis protein	N	2.82	Yes	No	No
CC_0164	Chain length regulator (capsular polysaccharide biosynthesis)	D	2.18	No (*)	No	No
CC_0556	Catalase	NC	2.00	No	No	No

Genes with reduced or increased expression in the Δ*sigT* strain upon 15 min of carbon starvation, with respect to the NA1000 wild type strain under the same conditions. SigT-dependency under osmotic stress is according to [28]. Upregulation by exposure to multiple heavy metals corresponds to genes upregulated by two or more metals according to [29]. Genes annotated as No(*) in the ‘sigT motif’ column correspond to cases where the sigT motif is found upstream of an adjacent gene as part of a putative operon (CC_0284 and CC_0285; CC_1179 and CC_1178; CC_0162, CC_0163 and CC_0164). COG groups: K = Transcription; T = Signal transduction mechanisms; E = Amino acid transport and metabolism; Q = Secondary metabolites biosynthesis; P = Inorganic ion transport and metabolism; S = Function unknown; R = General function prediction only; M = Cell wall/membrane/envelope biogenesis; N = Cell motility; D = Cell cycle control; NC = Not classified.

The main functional groups represented in the putative SigT carbon starvation regulon are signal transduction and gene regulation (7 genes), and transport and metabolism (5 genes). The gene encoding the SigU ECF sigma factor has a SigT binding motif in its promoter, suggesting it is directly regulated by SigT. SigU is activated by SigT during cell cycle progression [18], upon osmotic stress [25], [28] and upon carbon starvation (this work).

In order to determine if any of the SigT-dependent genes lacking the conserved SigT promoter binding motif were regulated by a pathway involving SigU, we used microarrays to compare the carbon starvation-induced expression levels in a wild type strain (LS101) and a *sigU* null mutant strain (LS3547). Only one gene, CC_3466, encoding a hypothetical protein, appeared to be SigU-dependent under carbon starvation. This gene, which was also found to be SigT-dependent, does not have the conserved SigT motif in its promoter, consistent with its SigT dependence being, directly or indirectly through the function of the SigU transcription factor. The CC3466 transcript is cell cycle regulated [24], with a distinct peak at the swarmer-to-stalked cell transition. This pattern was also observed in the cell cycle microarray results of McGrath *et al.* [25]. CC_3466 is not reported to be SigT-dependent upon osmotic stress [28]. On the other hand, its expression is induced by exposure to chromate and dichromate heavy metals [29], incubation in minimal media vs rich media [30], as well as carbon limitation (compared to nitrogen limitation) [12]. The predicted amino acid sequence of CC_3466 (102 amino acids with a predicted molecular weight of 11 KDa) yielded very few low scoring BLASTP hits outside of the *Caulobacter* genus. In our proteome analysis, peptide levels for CC_3466 increased significantly after 60 min of carbon starvation.

#### Carbon starvation CtrA regulon

The gene cluster sharing motif cc_8 was shown to be enriched in genes that were upregulated upon carbon starvation: five out of nine genes with this motif increased significantly upon 30 min of carbon starvation. Motifs cc_08, cc_10 and cc_11 share similarity with the binding motif for the CtrA cell cycle master regulator. While genes with motifs cc_10 and cc_11 showed an expression peak at the predivisional stage, genes with motif cc_8 showed an earlier peak, at the swarmer to stalk cell transition [25]. The CtrA regulon has been characterized by chIP-chip analysis, identifying genomic regions where CtrA binds, and microarray analysis, identifying genes with affected expression in a CtrA temperature sensitive mutant strain [31]. We cross-correlated these results with our carbon starvation microarray dataset and found that the expression of a significant number of both cell-cycle regulated and non cell-cycle regulated CtrA targets was altered upon 30 min of carbon starvation (Table 2). Among these targets was the CC_2644 gene, whose transcript was one of the most strongly upregulated transcripts upon carbon starvation. This gene, which remains uncharacterized in *C. crescentus*, encodes a protein belonging to the PhoH family. The *E. coli phoH* gene, which defines the family, was shown to be induced by phosphate starvation and to have ATPase activity [32].

**Table 2:** CtrA targets that change significantly upon carbon starvation.

Gene	Annotation	Transcript levels carbon starvation/control	Cell cycle regulated?
CC_2165	division plane positioning ATPase *mipZ*	5.88	Yes
CC_2540	Cell division protein *ftsZ*	2.15	Yes
CC_2063	Flagellar basal-body rod protein *flgF*	2.31	Yes
CC_2316	Transcriptional regulator	2.18	Yes
CC_3219	Two-component sensor histidine kinase	2.54	Yes
CC_2644	*phoH* protein	44.56	Yes
CC_1396	Lactate 2-monooxygenase	6.28	Yes
CC_1101	Protoporphyrinogen oxidase	2.37	Yes
CC_1307	Aspartyl protease *perP*	3.05	Yes
CC_1211	Hypothetical protein	2.01	Yes
CC_3291	Hypothetical protein	9.90	Yes
CC_1072	Ribonuclease H	3.35	Yes
CC_3218	Hypothetical protein	2.12	Yes
CC_1872	peptidoglycan-specific endopeptidase, M23 family	0.30	Yes
CC_0484	peptidyl-tRNA hydrolase	0.41	Yes
CC_1923	SSU ribosomal protein S2P	0.05	Yes
CC_3202	SSU ribosomal protein S12P	0.04	Yes
CC_2166	pantoate-beta-alanine ligase	0.37	Yes
CC_0050	S-adenosylmethionine synthetase	0.02	Yes
CC_0350	hypothetical protein with pentapeptide repeats	0.48	Yes
CC_3454	Acyl-CoA dehydrogenase	4.47	No
CC_0651	Conserved hypothetical protein	2.21	No
CC_0696	GumN superfamily protein	4.72	No
CC_1106	Ice nucleation protein	2.30	No
CC_2869	Hypothetical protein	2.32	No
CC_2966	3-oxoacyl-[acyl-carrier protein] reductase	3.45	No
CC_1892	aspartyl-tRNA synthetase	0.09	No
CC_2451	DNA topoisomerase I	0.11	No
CC_3155	chemotaxis receiver domain protein cheYIII	0.39	No
CC_2241	heat shock protein *hsp33*	0.30	No
CC_1034	GTP-binding protein *lepA*	0.30	No
CC_0249	SCO1/SenC family protein	0.46	No

Genes that change significantly upon 30 min of carbon starvation, and that are directly regulated by CtrA [31], and are either cell cycle regulated and non cell cycle regulated [24].

We performed the same enrichment analysis with the motifs identified by McGrath *et al.* for genes responding to heavy metal stress [25]. Sets sharing motifs m_2 (corresponding to the RpoD binding motif), m_3 (similar to motif cc_13) and m_4 were enriched in genes downregulated upon 30 min of carbon starvation, while sets sharing motifs m_5 (similar to motif cc_1) and m_13 were enriched in genes upregulated upon 30 min of carbon starvation. Since none of the additional sets had a known cognate regulator, this analysis did not identify further pathways related to the carbon starvation response.

### Cell-stage specific response to carbon starvation

**Figure 3:**
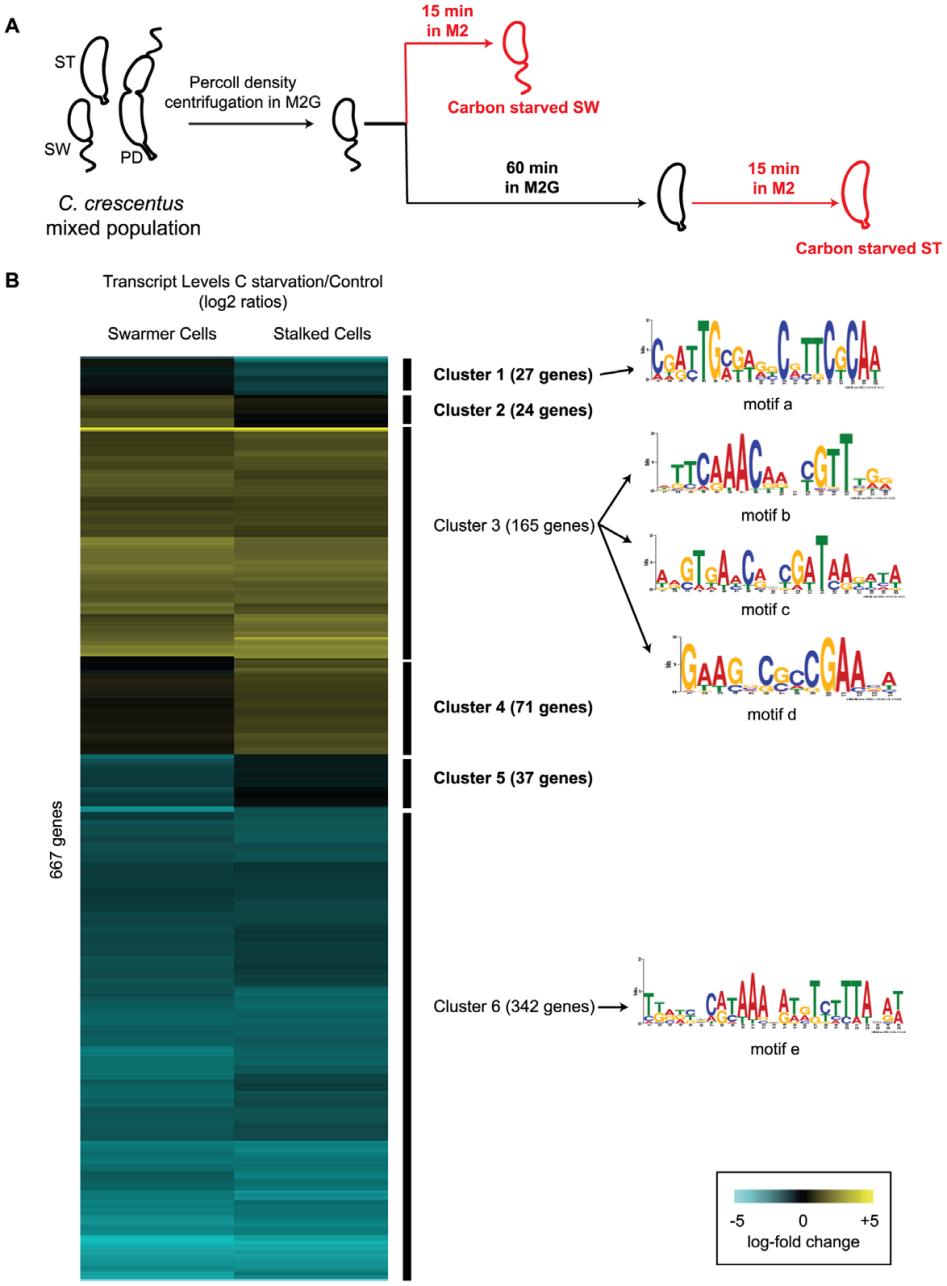
Cell stage-specific carbon starvation response. **A**. Diagram of the experimental design to explore the cell-stage specific response to carbon starvation. Isolated swarmer (SW) cells were subjected to glucose starvation for 15 min. To assess the response at the stalked cell stage (ST), swarmer cells were allowed to differentiate into stalked cells in complete minimal media for 60 min, and then subjected to glucose starvation for 15 minutes. At the indicated times, cell samples were collected and RNA extracted and transcribed to cDNA to hybridize onto *Caulobacter* microarray chips. PD = predivisional cell. M2 and M2G media are described in the Methods section. **B**. Hierarchical clustering of the transcriptional response to carbon starvation in swarmer cells and stalked cells. The values plotted are the log2-fold change ratios of the cells subjected to 15 minutes of carbon starvation and the non-starved controls. The promoter regions of the genes in each cluster (from −200 to +50 with respect to the translational start site) were used as input in the search for shared motifs using MEME. The five motifs with significant E-values and information content are shown.

We explored the response to carbon starvation separately in swarmer and stalked cell populations. We isolated swarmer cells and incubated them in M2 medium lacking glucose for 15 min before collecting samples (Figure 3A). To study the stalked cell response to carbon starvation, a population of swarmer cells was allowed to proceed through the cell cycle for 60 min and transition into stalked cells in complete medium, before removing the carbon source and incubating for 15 min in M2 medium. RNA was extracted from starved swarmer cells and starved stalked cells, and hybridized to the CauloHI1 chip. To account for changes in transcript levels normally occurring as the cell cycle progresses, we used the normalized values corresponding to 15 min and 75 min of the cell cycle microarray profiles obtained from the data of McGrath *et al.* [25], as the non-starved controls for swarmer and stalked cells, respectively. The log-2 values of the expression ratios of the starved and non-starved cells for both stages were used to determine the cell-stage specific responsiveness of each gene, and clustered accordingly (Figure 3B and Table S5). Six clusters were identified. Most of the genes showed a similar expression change in swarmer and stalked cells: out of 667 genes, 165 were upregulated (cluster 3), and 342 genes were downregulated (cluster 6), in both stages. The remaining clusters displayed a stage-differential response to carbon starvation: 27 genes did not change significantly in swarmer cells, but were down-regulated preferentially in stalked cells (cluster 1); 24 genes were upregulated in swarmer cells, but did not change significantly in stalked cells (cluster 2); 71 genes did not change significantly in swarmer cells, but were upregulated in stalked cells (cluster 4); 37 genes were down-regulated in swarmer cells, but did not change significantly in stalked cells (cluster 5). Using MEME motif finder [33], we identified five novel conserved DNA motifs in the upstream regions of genes corresponding to three of the clusters (motif a in cluster 1; b, c and d in cluster 3; and e in cluster 6).

### Adaptive changes of swarmer cells subjected to carbon starvation

**Figure 4:**
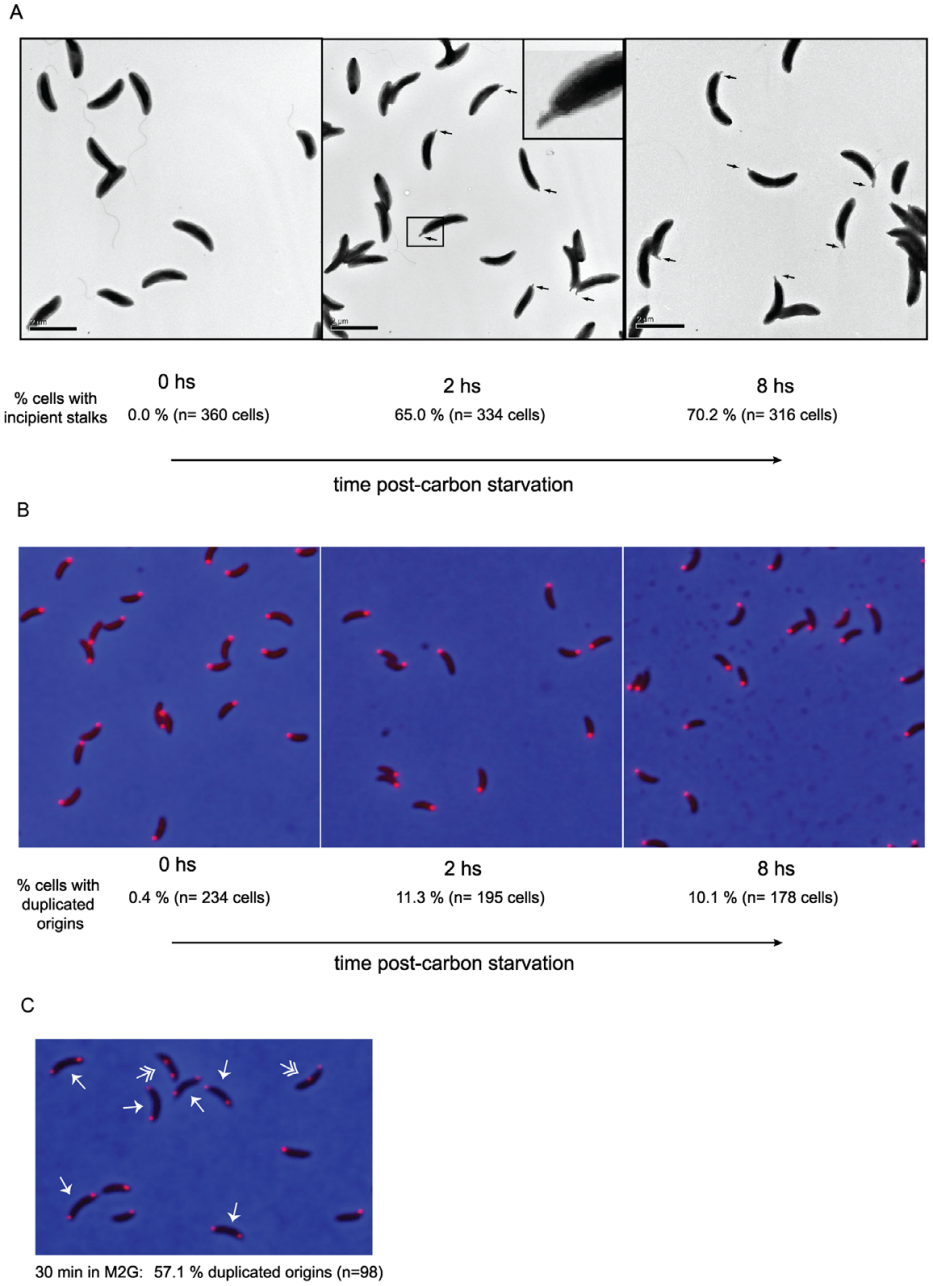
Adaptive changes of swarmer cells upon carbon starvation. **A**. Polar morphogenesis of wild type swarmer cells subjected to carbon starvation. Swarmer cells were incubated in M2 medium in the absence of glucose for 0, 2 and 8 hs, and visualized by electron microscopy, as described in Methods. The number of cells with incipient stalks (indicated by arrows and detailed in the inset in middle panel) was tallied for several fields and the corresponding percentage is indicated for each time point. **B**. Replication initiation in carbon starved swarmer cells was observed in a strain in which the *parB* gene was replaced with an *ecfp-parB* fusion, treated as described in A. At the indicated times, a sample was removed from the cultures, transferred unto an agarose pad an imaged. The number of cells with duplicated ECFP-ParB foci was counted for each time point and the percentage is indicated. **C**. Swarmer cells isolated for the experiment described in B were incubated in complete M2G media and imaged after 30 min. The percentage of cells with duplicated ECFP-ParB foci is indicated. Arrows indicate cells that have completed origin segregation, as evidence by ECFP-ParB foci in opposite poles, while double arrows indicate cells in the process of segregation.

In *Caulobacter*, the chromosomal replication origin is positioned at the cell pole, and upon initiation of replication, a copy of the origin sequence is moved to the opposite cell pole. Therefore, we can follow the onset of DNA replication by following the cellular position of the origin bound to the ParB segregation factor. In order to connect the stage-specific response to carbon starvation observed at the molecular level with cell cycle and differentiation events, we obtained transmission electron microscope images of swarmer cells immediately following their isolation, and then after subjecting them to carbon starvation. We examined the replication and segregation of the chromosomal origin (*ori*) locus, under the same conditions, using epifluorescence imaging of a strain carrying a fluorescently-tagged version of the ParB centromere-binding protein. 65% of the isolated swarmer cells developed short incipient stalks when incubated in the absence of glucose for 2 hs (Figure 4A). After 8 hs of starvation, these stalks failed to elongate. Under the same conditions, only about a tenth of the population had duplicated the chromosomal *ori* locus (Figure 4B), whereas after 30 min in complete media, 57% of the swarmers showed fully or partially segregated origins (Figure 4C). Thus, for a significant percentage of the population, carbon starvation uncoupled the initiation of stalk biogenesis and the G1-to-S transition, events that occur coincidently when nutrients are sufficient.

### SigT-dependent degradation of CtrA in swarmer cells upon carbon starvation

**Figure 5:**
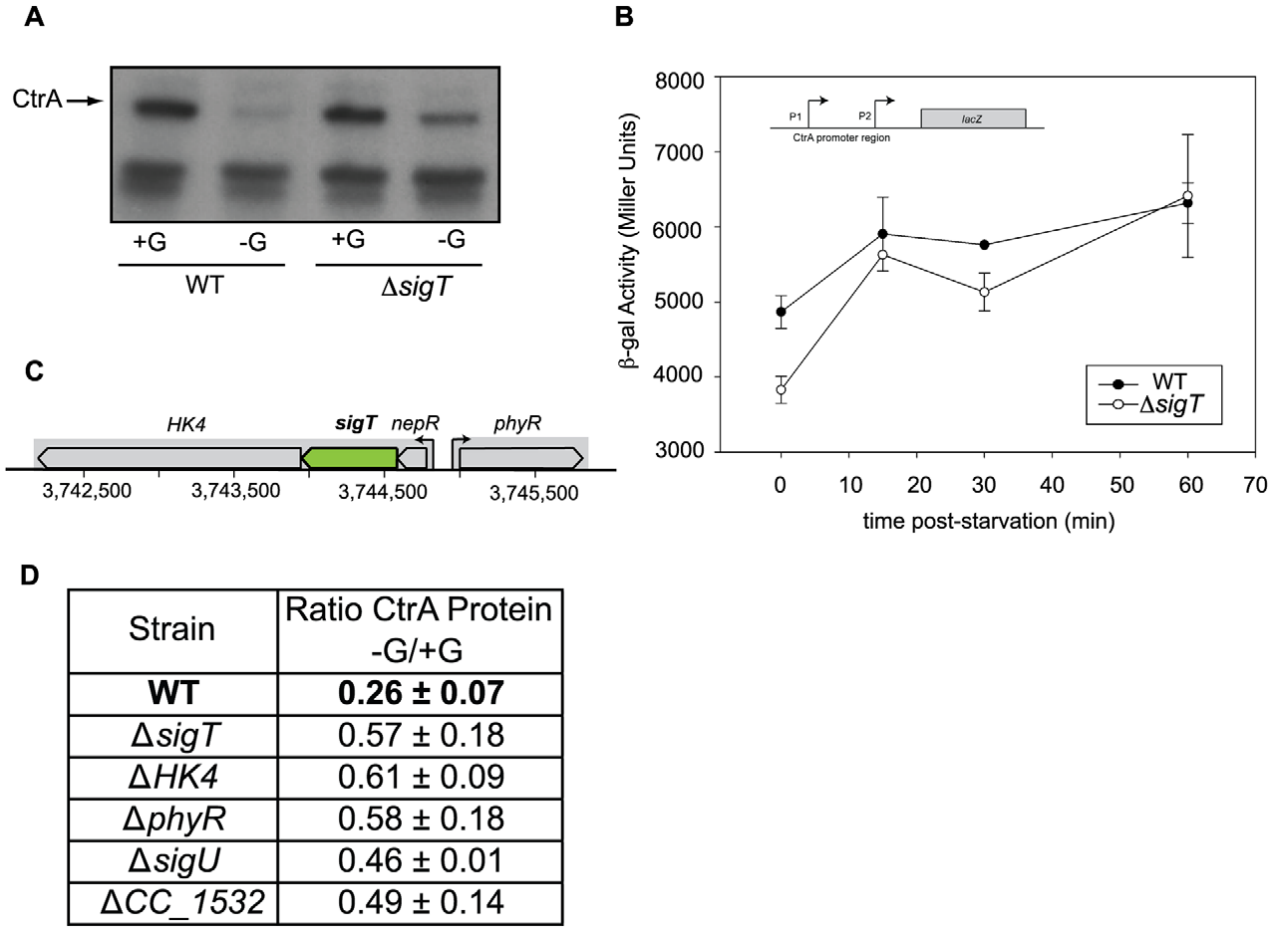
SigT-dependent degradation of CtrA upon carbon starvation. **A**. Levels of CtrA protein in swarmer cells in the presence and absence of a carbon source, in wild type cells and cells carrying a *sigT* deletion. Isolated swarmer cells from both genetic backgrounds were incubated in M2 medium in the absence or presence of 0.2% glucose. After 150 min, samples from these cultures were subjected to immunoblot analysis with an anti-CtrA polyclonal antibody. The band corresponding to CtrA is indicated with an arrow. **B**. A construct with the complete CtrA promoter region fused to a promoterless *lacZ* reporter in pRKlac290 was introduced into wild-type and *sigT* deletion strains. ß-galactosidase activity was measured in swarmer cells starved for carbon for up to 60 min. **C**. Genomic context of *sigT*. The coordinates correspond to the *C. crescentus* NA1000 genome. CC_3474 (HK = Histidine Kinase) corresponds to CCNA_3588 in NA1000; *sigT* corresponds to CC_3475 (CCNA_3589); *nepR* corresponds to CC_3476 (CCNA_3590); *phyR* corresponds to CC_3477 (CCNA_3591). **D**. Relative levels of the CtrA protein in the presence and absence of carbon, assayed as described in A, are shown for different mutant backgrounds, including deletions of genes in the genomic region of *sigT*, and of SigT-dependent genes (see Table 1). +G corresponds to M2 medium in the presence of 0.2% glucose; -G corresponds to M2 in the absence of glucose. The sampling times are 150 min after starvation, as in A. The error for the ratios corresponds to the standard deviation of the mean for at least two experiments.

The CtrA cell cycle master regulator is a critical element of the core machinery that regulates cell cycle progression. CtrA binds to the chromosomal origin of replication and blocks replication initiation in the swarmer cell [34]. Upon differentiation of the swarmer cell to the stalked cell, CtrA is cleared from the cell by proteolysis, allowing the initiation of DNA replication. CtrA is re-synthesized following replication initiation. Our proteome studies showed a decrease in the protein levels of the CtrA master regulator in a mixed population of *Caulobacter* cells subjected to carbon starvation: CtrA levels in cells incubated in the absence of glucose for 30 min were half of that of cells incubated in the presence of glucose (Table 2). We observed a similar decrease in the relative levels of CtrA by immunoblot assays of carbon starved mixed cell populations (not shown) and swarmer cells, as previously reported [10]. Swarmer cells incubated for 150 min in the absence of glucose showed low levels of the CtrA protein, while those incubated for 150 min in the presence of glucose had progressed through the cell cycle and reaccumulated CtrA. In contrast, when the same assay was performed with swarmer cells carrying a *sigT* deletion, a less significant decrease in CtrA levels was observed in the absence of glucose, indicating that SigT contributes to the clearance of CtrA protein in carbon starved swarmer cells (Figure 5A and D). We measured the activity of the CtrA promoter under carbon starvation in a wild type and *sigT* deletion background, using a transcriptional fusion of the CtrA promoter region to *lacZ*. We found only a minimal difference in the transcriptional activity of the reporter in both backgrounds (Figure 5B). Further experiments will be carried out to determine if SigT affects the starvation-induced clearance of CtrA at the post-transcriptional level.

Since SigT is a transcriptional regulator, the simplest explanation for these results is that a downstream target whose expression is SigT-dependent is responsible for the effect on CtrA protein stability. Consequently, we compared the starvation and non-starvation levels of CtrA (as described for Figure 5A) in different deletion mutant backgrounds for selected SigT carbon starvation targets identified in our microarray analysis. We also assayed mutants carrying deletions in genes that are colocated with *sigT* in the chromosome (Figure 5C) and are presumed regulators of its activity. As shown in Figure 5D, deletions in both ECF Sigma factor *sigU* and the conserved hypothetical protein CC_1532 showed levels of CtrA stabilization that are similar to those observed for the *sigT* deletion strain, suggesting they participate in the same pathway that leads to CtrA degradation under starvation. This is also the case for the genes encoding the histidine kinase HK4, and the response regulator PhyR (CC_3473), that was recently postulated to function as an anti-anti-sigma factor for SigT (or homologous sigma factor) in *Methylobacterium extorquens* [35], *Sinorhizobium meliloti* [36] and *C. crescentus* [37]. In this assay, we did not test *nepR*, the postulated anti-sigma factor for SigT [37].

## Discussion

When faced with nutrient limitation, bacterial cells must deploy an array of scavenging systems, adapt their metabolic fluxes to compensate for missing compounds, and limit energy-consuming growth and cell division processes. *C. crescentus* is an oligotroph whose physiology is adapted for survival in environments characterized by low and fluctuating nutrient levels [1]. Here we have examined the response of *C. crescentus* to the sudden onset of carbon starvation.

### Carbon starvation uncouples swarmer cell differentiation and the G1-to-S phase transition

In the presence of adequate nutrient levels, the swarmer-to-stalked-cell transition involves the loss of the polar flagellum, the biogenesis of a stalk in its place, and the concurrent initiation of chromosome replication. It has been shown however, that these processes can be uncoupled. When the DnaA activator of DNA replication is depleted [21], or when CtrA inactivation is prevented [38], swarmer cells undergo polar morphological changes, but fail to initiate chromosome replication. Meanwhile, swarmer cells carrying a deletion in the gene encoding the PleD response regulator replicate their chromosome in the absence of polar morphogenesis [39]. We show here that wild type swarmer cells subjected to abrupt carbon depletion were capable of initiating, but not completing, stalk development (see Figure 4A), while the replication and segregation of the chromosomal origin was inhibited (see Figure 4B, [10]). Because the incipient stalks could not be detected by light microscopy, it was previously postulated that carbon starvation inhibits swarmer differentiation. Furthermore, in these experiments cell populations were starved for carbon prior to isolation of swarmer cells [10]. However, electron microscope images (see Figure 4A) revealed the presence of incipient stalks under these conditions. Thus, the point of commitment to initiate stalk morphogenesis appears to precede that of initiation of chromosomal replication. In the population of swarmer cells obtained by the synchronization procedure, the younger cells that are most recently derived from cell division might block both processes, accounting for the 30% of cells that did not develop an incipient stalk by 8 hs of carbon starvation. On the other hand, the majority of swarmer cells in the population that had already committed to polar morphogenesis, blocked the initiation of chromosome replication. Since the stalk is associated with the ability to scavenge for nutrients [15], the initiation of stalk development upon sustained carbon starvation might prime the cell for quick resumption of cell cycle progression once nutrients become available.

### Transcript and protein changes in the response to carbon starvation

Transcript profiles as a function of the cell cycle have revealed just-in-time transcriptional activation of distinct functional modules in *C. crescentus* [24]. However, multiple layers of post-transcriptional regulation are known to be involved in the complex orchestration of cell cycle progression and polar differentiation in this bacterium (see [13], [40] for reviews). For this reason, our global analysis included not only the transcriptome but also the proteome profile of *C. crescentus* cells subjected to carbon starvation.

Table 3 shows previously characterized proteins of interest from selected functional groups, that change significantly upon 30 and 60 min of carbon starvation. The levels of FtsZ, essential for cytokinesis in *C. crescentus* [41], and FtsX, a predicted ABC transporter that is needed for cell division in *E. coli* [42], decrease significantly upon carbon starvation. This is consistent with the inhibition of cell division that occurs upon carbon starvation.

**Table 3:** Proteins of interest in selected functional categories that change upon carbon starvation.

Functional category	Upregulated proteins	Downregulated proteins
Cell wall/membrane/envelope biogenesis (COG M) and cell division	TolA, TolB, TolR, TolQ, BamA, BamB, BamE, BamD, DipM, MreC	FtsZ, FtsX
Cell cycle control		CtrA, DnaA, GcrA
Post-translational modification (COG O)	ClpA, FtsH, SppA	DnaJ, Hsp33
Signal transduction and gene regulation	DivL (HK), FixL (HK), CckA (HK-RR)	FlbD (RR), PleD (RR), DivK (RR), McpA, CheAI, CheYI, CheD
Chromosome structure and dynamics	Smc	PopZ, ParA

The classification is based upon COG (NCBI) and the literature. Abbreviations: HK = histidine kinase; RR = response regulator; HK-RR = hybrid histidine kinase-response regulator.

Proteases have traditionally been associated with the response to environmental stress, as cells need to re-engineer the cellular landscape, recycle damaged and unwanted proteins, and selectively target key regulators [43]. The levels of two major proteolysis-related factors increased significantly upon carbon starvation, and could be fulfilling this role in *C. crescentus*: the FtsH protease, previously characterized as a component of *C. crescentus'* general stress response [44], and the ClpA chaperone, which normally works in concert with the ClpP protease, and has not been thus far associated with the stress response.

The level of Smc (CC_0373), a nucleoid-associated protein required for chromosome structure maintenance and segregation in *C. crescentus* [45], increased significantly upon carbon starvation. This raises the possibility that the nucleoid of growth-arrested cells adopts a different compaction state than that in exponentially growing cells, possibly contributing to the inhibition of DNA replication and segregation, as well as an increased tolerance to stress-related damaging agents. *E. coli's* nucleoid protein composition has been shown to be growth phase-dependent [46] and a factor in long term survival [47].

Respiratory metabolism generates reactive oxygen species that may damage membranes, DNA and proteins. As long as the environment promotes growth and continued de novo synthesis, the oxidized macromolecules are rapidly diluted, but this is not the case during growth-arrest of metabolically active cells. Correlation of our carbon starvation datasets with microarray analysis of *C. crescentus'* response to metal stress [29], revealed that of 222 genes that were induced by two or more of the four heavy metals tested, 38 were upregulated in our carbon starvation proteomics samples, and 103 were induced after 30 minutes of carbon starvation at the transcript level (Table S6). These results suggest that a subset of the genes that respond to carbon starvation are part of a general stress response. In *E. coli* and *B. subtilis*, growth arrest caused by starvation has also been shown to elicit increased synthesis of proteins normally induced by oxidative stress, and starved cells display cross-resistance to these stresses [7], [48], [49].

**Figure 6:**
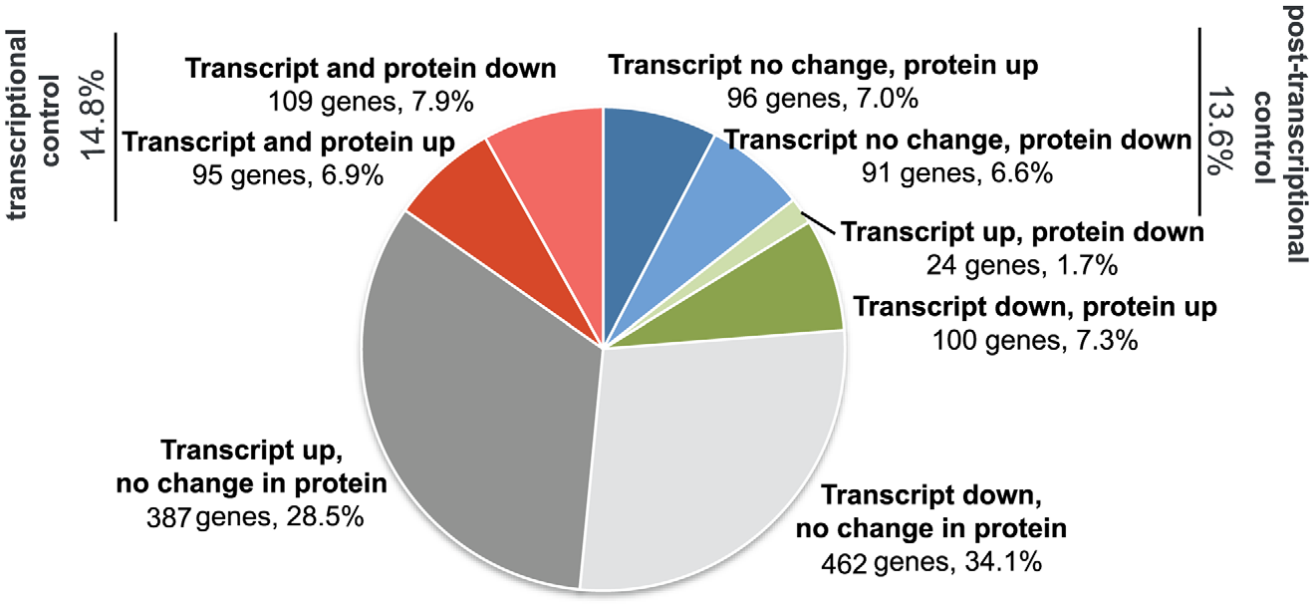
Distribution of carbon starvation-induced changes at the transcript and protein levels. For the 1364 genes for which we had both microarrays and proteomics valid data, and a significant change had been observed for at least one of the datasets, the intersection and exclusion sets are represented. Table S7 lists the genes that belong to each group in the distribution.

The correlation of the set of transcripts and the set of proteins that change significantly upon starvation revealed interesting insights regarding the levels of regulation that might be operating in the carbon starvation response (Figure 6 and Table S7). Considering the 1364 genes for which proteome data were obtained and there was a significant change upon carbon starvation at either the transcript or protein level, or both, in more than half of the cases, the change was only observed at the transcript level (28.5% of genes were up-regulated and 34.1% were downregulated). Some of these might reflect an inherent greater sensitivity of the microarray technique used for detection of transcript changes, than that of the mass spectrometry method used to probe changes in protein levels. 14.8% of the genes showed changes in the same direction at both the transcript and protein level, consistent with transcriptional regulation. For a similar proportion of genes (13.6%), no change was observed at the transcript level, but the corresponding protein was significantly up- or down-regulated. This group contains candidates for post-transcriptional regulation, either at the translation or protein stability levels. For 9.0% of the genes, observed changes at transcript and protein levels occurred in opposite directions. Overall, our observations are in line with a complex interplay of regulatory mechanisms operating in bacteria, and the lack of correlation between mRNA and protein levels observed in *E. coli* [50], [51].

### Regulatory networks controlling *C. crescentus'* response to carbon starvation

**Figure 7:**
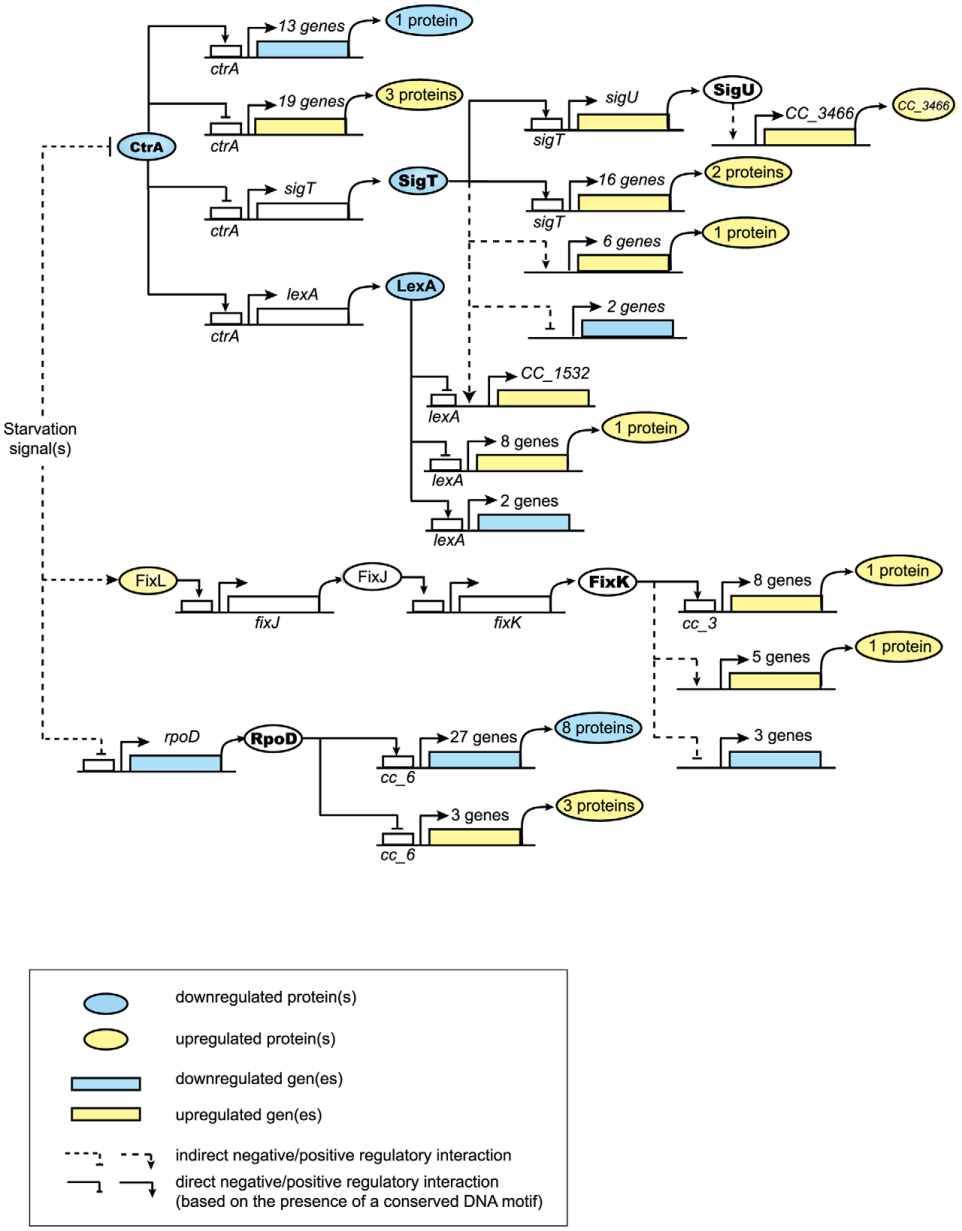
Carbon starvation regulatory pathways. Diagram of regulatory pathways involved in the response to carbon starvation, derived from the analysis of proteomic and gene expression profile changes. Ovals represent proteins and rectangles represent genes. Genes and proteins whose levels increase significantly upon starvation are represented in yellow, and downregulated genes and proteins in blue. For example, we indicate that of the 13 genes controlled by CtrA whose transcripts were downregulated in the absence of carbon (blue rectangle), we were able to detect the down-regulation of one of the corresponding protein products (blue oval). The same schematic representation was used for all genes shown. The genes and proteins that change significantly upon carbon starvation are listed in Table S8. Putative direct regulatory interactions are based on the presence of conserved promoter sequences, while an indirect regulation is proposed if there is no evidence of DNA binding or of the presence of a conserved promoter element. For the transcript changes, the 30 min time point was considered, while both the 30 and 60 min time points were considered for the protein changes. The CtrA regulon comprises direct CtrA targets, as previously determined [31], that changed upon 30 min of carbon starvation determined by microarray analysis (listed in Table 2), as well as proteins encoded by direct CtrA targets that changed in the proteomic analysis at 30 and/or 60 min of carbon starvation (while most of those proteins were seen to change at the transcript level as well, SigU and LexA were observed to change only at the protein level). CtrA activation or repression was inferred by the direction of the change observed upon starvation: those genes with increased transcript levels upon starvation were inferred to be negatively regulated by CtrA (and consequently derepressed as CtrA protein levels decrease upon starvation); genes with decreased transcript levels were inferred to be positively regulated by CtrA. The SigT regulon includes the genes that showed a significant difference in transcript levels changes upon 15 min of carbon starvation in the *sigT* deletion strain with respect to wild type (listed in Table 1). Genes with a SigT binding motif in their promoters are represented as direct targets, while those lacking the motif, as indirect targets. The levels of proteins encoded by a subset of these genes changed significantly upon 30 and/or 60 min of carbon starvation. The SigU-dependent gene, CC_3466, showed reduced transcript levels under carbon starvation in a *sigU* deletion strain with respect to wild type. The LexA (SOS) regulon comprises those genes belonging to the SOS regulon –as previously determined [52] - that changed significantly after 30 min of carbon starvation. The topology of the FixL-FixJ-FixK pathway is as determined by Crosson *et al.* [27]. The FixK direct targets are those that have the cc_3 motif in their promoter, while the indirect targets were shown to be FixK-dependent by Crosson *et al.*, but lack the cc_3 motif. The RpoD regulon comprises the genes with the cc_6 promoter motif whose transcripts levels were upregulated or downregulated upon 30 min of carbon starvation.

Analysis of transcriptional profiles allowed us to identify regulators and regulatory modules involved in the response to carbon starvation and, more specifically, connections with the regulatory network that controls cell cycle progression. The enrichment analysis based on previously characterized clusters of co-expressed cell cycle genes that share conserved promoter motifs [25], indicated that the RpoD housekeeping sigma factor, the FixK transcription factor, the SigT ECF sigma factor, and the CtrA cell cycle master regulator play significant roles in the response of *C. crescentus* to carbon starvation. The diagram shown in Figure 7 integrates our transcriptome and proteome datasets of the response to carbon starvation, with regulatory pathways derived from microarray data published for CtrA [31], FixK [27], and LexA [52] (See Tables 2 and S8). The pathways for SigT and SigU under starvation conditions were inferred from data presented in this paper (See Table 1). The proposed transcriptional regulatory interactions were assumed to be direct when the cognate DNA binding motif for the regulator was located in the promoter region of the target gene.

#### RpoD

The transcript levels of housekeeping sigma factor RpoD and 30 genes identified as RpoD targets by the presence of its cognate DNA motif (cc_6), changed significantly upon carbon starvation, the great majority of them being downregulated. Five of the proteins predicted to be encoded by these genes also changed significantly upon carbon starvation (Figure 7 and Table S8). A decrease in RpoD protein levels was not observed under these conditions, pointing to an alternative mechanism of regulation of its activity that would explain the down-regulation of its target genes, such as the competition from alternative sigma factors induced upon starvation, in binding to the core RNA polymerase.

#### FixK

The FixL-FixJ-FixK pathway was shown to be a major component of *C. crescentus'* response to hypoxia by Crosson *et al.* [27]. Our analysis of the response to carbon starvation revealed that the protein levels of sensor histidine kinase FixL increased, while 16 of the genes directly (bearing the motif cc_3 in their promoter regions) or indirectly regulated by transcriptional regulator FixK, changed significantly upon carbon starvation (Figure 7). Two proteins encoded by these FixK-regulated genes were observed to be up-regulated under the same conditions. Interestingly, only half of the genes that have the cc_3 motif were identified as part of the hypoxia-induced FixK regulon by Crosson *et al.*, suggesting that FixK might activate different sets of genes in response to different environmental stimuli or that additional regulatory pathways or factors are involved in these responses.

#### CtrA

Out of 55 cell cycle-regulated CtrA target genes, we found that 13 were upregulated and 7 were downregulated after 30 minutes of carbon starvation. Out of 32 non-cell cycle-regulated CtrA target genes, 6 were upregulated and 6 were downregulated after 30 minutes of carbon starvation (Table 2 and Figure 7). Our proteome analysis showed a significant decrease in the protein level of CtrA in response to carbon starvation, consistent with previous analyses [10], [22]. Of the CtrA-regulated cell cycle transcripts [25] that were upregulated upon carbon starvation (Table 2), 11 out of 13 peaked at the stalked or predivisional stage, while the levels of 10 of these 13 transcripts were reported to increase in a strain carrying a temperature sensitive allele of CtrA (CtrA^ts^) incubated at restrictive temperature [31]. These observations are compatible with these genes being de-repressed as CtrA protein levels drop in swarmer cells starved for carbon. Conversely, 5 out of the 7 CtrA cell cycle target genes that are down-regulated in response to carbon starvation, show lower transcript levels at the swarmer stage of the cell cycle, with 2 of them displaying increased levels in the CtrA thermosensitive mutant at the restrictive temperature. These genes are most likely deactivated in carbon starved swarmer cells as CtrA levels drop. The remaining 12 CtrA target genes, whose transcript levels remain constant during cell cycle progression in complete media, point to a novel role for CtrA in activating and repressing genes involved in the response to starvation and other stress signals, in addition to those involved in cell cycle progression.

#### LexA

Upon carbon starvation, the protein levels of LexA –a CtrA target- were observed to decrease to 60% of the non-starvation levels (although the p-value (0.09) didn't meet the cutoff established for significance (0.05)). The SOS response, the prototypical response to DNA damage in prokaryotes, is controlled by the opposing activities of the LexA repressor and the RecA activator proteins [53]. Genes in the SOS regulon, repressed by LexA under basal conditions, are activated in response to single-stranded DNA regions, often as result of DNA replication inhibition [54]. Activation of the SOS response in *E. coli* leads to blocked FtsZ ring formation and cell division, via the SulA protein [55]. It is possible that a LexA target interacts with FtsZ in *C. crescentus*, to mediate the cell division arrest observed upon starvation [10]. Out of the 44 previously characterized direct LexA target genes [52], two were downregulated and nine were upregulated upon 30 min of carbon starvation, consistent with the decrease in LexA protein levels; one of the upregulated genes, encoding the CC_2589 hypothetical protein, was also upregulated at the protein level.

### SigT is a regulator of the starvation-stress response in *C. crescentus* and is involved in the starvation-induced degradation of the CtrA master regulator

Alternative sigma factors play key roles in various stress responses and morphological differentiation in bacteria. Upon activation by environmental or internal signals, alternative sigma factors direct RNA polymerase promoter specificity to activate different regulons. In most gram-negative bacteria, the transcriptional response to environmental stresses is dominated by alternative sigma factor RpoS (see [56] for a review). In gram-positive bacteria, that role falls upon the alternative sigma factor SigB (see [57] for a review). However, the α-proteobacteria lack homologues of either of these sigma factors [8], [58].

Three of the 13 *C. crescentus* ECF alternative sigma factors have been implicated in the response to specific stress conditions: SigF mediates the response to oxidative stress in stationary phase [59], SigE mediates the response to cadmium, organic hydroperoxide, singlet oxygen and UV [60], and SigT mediates the response to osmotic and oxidative stress [28]. Our results support a broader role for SigT. The 27 genes that respond to carbon starvation in a SigT-dependent manner comprise the carbon starvation SigT regulon (Table 1 and Figure 7). Some of these genes were also found to change at the protein level, including CC_3466 (Table 1, Table S2), which appears to be regulated through the SigU sigma factor. Moreover, there is an overlap of the SigT carbon starvation regulon with both the SigT osmotic stress regulon [28] (10 genes in common between the two sets), and the set of genes that are activated by exposure to several heavy metals [29] (12 genes in common). These observations suggest that a core set of SigT regulated genes belongs to a general starvation-stress response. Members of SigT's subfamily of ECF sigma factors from *Sinorhizobium meliloti* and *Methylobacterium extorquens* have been implicated in the regulation of the general stress response [35], [61]. Interestingly, some of the transcripts encoding regulatory proteins that were shown to be SigT-dependent upon carbon starvation -namely CC_1356, CC_0284 and CC_1178- required RpoN for induction in carbon versus nitrogen-limited conditions [12]. Two of these genes, CC_1356 and CC_0284 were reported to be SigT-dependent under conditions of osmotic stress [28].

The *sigT* gene belongs to the CtrA cell cycle regulon. CtrA is a negative regulator of *sigT* expression, and *sigT* transcripts peak when CtrA is cleared from the cell at the swarmer to stalked cell transition [31]. The transcript levels of *sigT* were not seen to change significantly upon carbon starvation at the sampled times, in either mixed population or isolated swarmer and stalked cells, while the SigT protein level was found to decrease after 60 minutes of carbon starvation. It is likely that SigT is regulated post-translationally. SigT belongs to the ECF sub-family (ECF15 or EcfG-like) that is characterized by a conserved genomic context, which includes the genes encoding the HK4 histidine kinase and the PhyR response regulator (see Fig. 5C), putative candidates to modulate the activity of the sigma factor [62]. A recent report postulates that *C. crescentus' sigT* is regulated by the anti-sigma factor NepR (CC_3476, cotranscribed with *sigT*), and the gene encoding PhyR, acting as an anti-anti-sigma factor, that is transcribed divergently from *sigT* [37].

**Figure 8:**
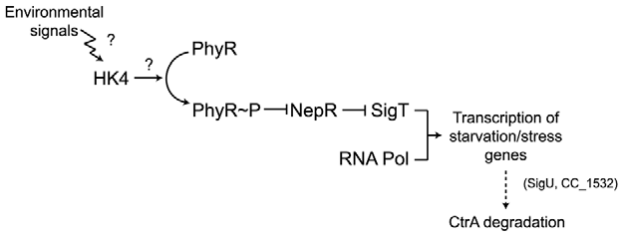
Diagram of the putative HK4-SigT signal transduction pathway. Shown is an inferred pathway based on our results, suggesting that SigT, HK4 (CC_3474), PhyR, SigU and CC_1532 contribute to the degradation of CtrA upon carbon starvation.

We propose that a SigT-dependent pathway is involved in the degradation of CtrA upon carbon starvation, and that this pathway includes the response regulator PhyR and the HK4 histidine kinase. The ability of the anti-anti-sigma factor PhyR to sequester the anti-sigma factor NepR, releasing this SigT to activate transcription, has been shown to be dependent on PhyR phosphorylation state [37]. It is possible that the HK4 histidine kinase, which possesses a predicted signal peptide that would target it to the membrane, is the protein responsible for sensing environmental cues and inducing a response in the CtrA starvation degradation pathway, via PhyR (Figure 8).

## Materials and Methods

### Bacterial strains and growth conditions

All strains were derived from wild type *C. crescentus* (NCBI Taxonomy ID: 155892) strain CB15N (NA1000) [63]. PYE medium (0.2% Bacto Peptone (Difco), 0.1% yeast extract (Difco), 1 mM MgSO_4_, and 0.5 mM CaCl_2_) was used to grow strains for cloning purposes. In all other cases, strains were grown in M2 minimal medium (6.1 mM Na_2_HPO_4_, 3.9 mM KH_2_PO_4_, 9.3 mM NH_4_Cl, 0.5 mM MgSO_4_, 10 µM FeSO_4_ (EDTA chelate; Sigma), 0.5 mM CaCl2) with 0.2% glucose as the sole carbon source (referred to as M2G). All *Caulobacter* strains were grown at 28°C and were not allowed to reach an OD_600_ higher than 0.4 at any time, to minimize differences due to physiological adaptation of cultures to stationary phase conditions. *Escherichia coli* strain TOP10 (Invitrogen) was used for cloning following standard procedures.

### Construction of gene deletion strains

In frame deletions of *sigT* (CC_3475), *sigU* (CC_2883), *phyR* (CC_3477), *HK4* (CC_3474), CC_3466 and CC_1532 were obtained by a two-step *sacB* counterselection procedure, as previously described [64].

### Culture synchronization to obtain isolated swarmer and stalked cells

Swarmer cells were isolated from mixed population cultures using a modified version of the percoll density centrifugation protocol [65]. Isolated colonies from a PYE plate were used to inoculate an M2G culture, grown to an optical density at 600 nm of 0.3. Cultures were cooled on ice, pelleted at 9,000×g at 4°C, and resuspended in 300 µl of ice-cold M2G. After adjusting the volume to 1 ml, one volume of ice-cold Percoll was added and mixed thoroughly. The suspension was centrifuged at 11,000×g for 20 min at 4°C, to separate the swarmer cells (lower band) from the rest of the population (top band). To obtain the stalked cells samples for the experiment in Figure 3, the synchronization procedure was performed as described, swarmers were resuspended in M2G, and allowed to differentiate for 60 min at 28°C. At that point, loss of motility of the majority of the population was confirmed by microscopy.

### Carbon starvation of wild-type swarmers and stalked cells, and of mixed populations of wild-type, Δ*sigT* and Δ*sigU* strains

Cells (swarmers or mixed populations, as indicated in each case) were washed twice in either ice-cold M2G (control samples) or ice-cold M2 (carbon starvation samples), and resuspended in 2 ml of pre-warmed (28°C) M2G or M2 media. Optical density was adjusted to ∼0.3–0.4. After 15 min of incubation at 28°C, cells were pelleted, frozen in liquid nitrogen, and transferred to −80°C. RNA isolation (see below) was always performed within 24 hs of obtaining the samples.

### Carbon starvation of *C. crescentus* mixed population for proteomics and microarrays assays

Cells were grown overnight in small cultures in M2G medium (never reaching OD_600 nm_ greater than 0.4). These cultures were used to inoculate 1200 ml cultures, which were incubated at 28°C until they had reached an OD_600 nm_ of 0.3–0.4. Cells were collected by centrifugation (5 min at 9,000×*g*) and washed twice with a large volume of ice-cold M2 medium, resuspending by gentle pipetting. Control cells were mock-washed with ice-cold M2 with 0.2% glucose. Washed cells were resuspended in pre-warmed M2 or M2G media and incubated at 28°C in a water bath with shaking. Samples were collected at 30 and 60 minutes, frozen in liquid nitrogen and stored at −80°C.

### Liquid chromatography-mass spectrometry analysis

Cells were pelleted, frozen in liquid nitrogen and stored at −80°C for no more than 2 days. Lysis and protein treatment was done as described previously [66]. Cell were lysed by bead beating. Proteins were denatured, reduced, alkylated, concentrated by solid-phase extraction, and digested with trypsin. For each injection, 1 µL of each sample of resuspended peptides was injected onto a reversed-phase column using an Isco LC system (Teledyne Isco) and eluted into a Thermo LTQ mass spectrometer (ThermoFinnigan, Inc., San Jose, CA). The mass spectrometer was operated in a data-dependent scan mode as previously described [67]. Raw data were analyzed with the Sequest program with an in silico database obtained from simulated tryptic digestion of the *C. crescentus* genome. Peptides from this analysis meeting our previously-used scoring standards [67] were used for abundance measurements. Peptide abundances were estimated from ion chromatograms using Viper [68]. Data were extracted and tabulated using SQL queries generated by a custom query from in-house databases. Data analysis, following the general normalization and rollup protocol described previously [69], was performed using the in-house quantitative proteomics tool DAnTE [70] and the statistical software package R (r-project.org). Recorded intensities were converted to natural logarithms throughout. Datasets consisted of the raw peptide intensities, reported as ion counts, for all experiments. All data sets were aligned together as a single batch using MultiAlign, and centered to the most complete data set using the *C. crescentus* accurate mass tag (AMT) database [68]. For subsequent work, peptide intensities were linear regressed to the median value of each experimental replicate data set. Normalized peptide intensities were rolled up into effective intensities for each protein in each experimental replicate using the RRollup algorithm [70]. For the binary comparisons of interest, the mean differential expression was calculated for each protein and the statistical significance of the differential expression was established using a two-sided t-test. Significance was established at p<0.05. To take into account very low abundance proteins, the counts of observed peptides under different treatment conditions were also compared using a standard Fisher's Exact Test on the 5 most abundant peptides. Proteins that showed significantly different peptide counts (p<0.05) were pooled together with the proteins that showed a significant change by intensity analysis, to report the final list of significant changes.

### Transmission electron microscopy

Cells were collected by centrifugation, fixed for 15 min at room temperature in 4% glutaraldehyde in 100 mM cacodylate-HCl buffer pH 7.4, washed, resuspended in cacodylate buffer, and preserved at 4°C until imaging. Fixed cells were spotted onto glow discharged formvar-carbon coated 300 mesh copper grids (Electron Microscopy Sciences), and allowed to settled for 1 min. Grids were then washed with two drops of mQ water, stained for 15 seconds with 1% uranyl acetate, washed again with two drops of mQ water and air dried. Grids were imaged at 80 kV on a JEOL TEM1230 system. Images were captured with a Gatan 967 slow-scan, cooled CCD camera, using the associated Gatan software. The numbers of cells with or without incipient stalks were counted manually from the exported images.

### Light and fluorescent microscopy

Swarmer cells from a strain carrying a *ecfp-parB* fusion in place of the *parB* gene in the chromosome (MT190) [71] were isolated, washed as described above to remove glucose, and immobilized onto freshly prepared 1% agarose-M2 pads onto microscopy slides, at the indicated times. Microscopy was performed on a DM6000B upright microscope (Leica) fitted with a 100× 1.46 NA HCX Plan APO oil immersion objective (Leica) and a Hamamatsu C9100 EM CCD camera. Phase contrast and fluorescent images (CFP channel, Ex 438/24 nm, Em 483/32 nm) were taken at 40 and 100 ms exposure times, respectively. Images were acquired using KAMS-acquire, a custom software program developed in-house [72], to control the microscope and camera. We used Photoshop CS4 (Adobe) to make false color merges of phase and fluorescent images. In order to tally cells with duplicated and non-duplicated origins, we processed KAMS-acquire files with a Matlab script developed in-house to obtain ordered images of individual cells (S. Hong, unpublished), which were then counted manually.

### Immunoblot analysis

Cell samples normalized by OD_600_ were lysed by boiling in 2× sample buffer (4% Sodium Dodecyl Sulphate (SDS), 20% glycerol, 0.01% Bromophenol Blue, 0.125 M Tris-HCl pH 6.8), and loaded in 8–16% Precise polyacrylamide gradient gels (Pierce), followed by electrophoretic transfer to a PVDF membrane (Millipore). Immunoblotting was done using anti-CtrA polyclonal serum (1∶10,000), and horseradish-peroxidase conjugated goat anti-rabbit IgG (1∶20,000). Signal was detected with chemiluminescence reagent (Perkin-Elmer) and BioMax MR film. The developed film was scanned, processed with Photoshop CS4 (Adobe), and band intensities were determined using ImageQuant (Molecular Dynamics).

### β-galactosidase assays

The β-galactosidase activity of a wild type and a Δ*sigT* strain carrying the plasmid pCtrA290 [73], with the CtrA promoter region fused to *lacZ*, was assayed after removal of glucose from the cultures in M2 medium, using o-nitrophenyl-β-D-galactoside (ONPG) [74].

### RNA Extraction, cDNA synthesis and processing, and microarrays hybridization

Total RNA was extracted from cells using the Purelink Mini Total RNA Purification System (Invitrogen), according to the manufacturer's instructions for on-column DNAse treatment to remove contaminating DNA. A maximum of 2 ml of culture of OD_600 nm_ between 0.3 and 0.4 was used per ml of Trizol. Integrity of the RNA was confirmed using the RNA 6000 Nano kit (Agilent) on a Agilent 2100 Bioanalyzer, and its concentration was calculated from OD_260 nm_ measurements on a nanodrop. cDNA was synthesized using Super Script II (Invitrogen) with random hexamers, according to the manufacturer's instructions. After reverse transcription, RNA was removed by 30 min NaOH incubation at 65°C and cDNA was purified using MinElute columns (Qiagen). Purified cDNA was fragmented by a 5 min incubation with DNAseI (Invitrogen; 0.6 U/µg cDNA) in a thermocycler, followed by 15 min enzyme inactivation at 98°C. DNAseI activity was previously titrated, and the same batch of enzyme and thermocycler were used with the same settings across all experiments to obtain reproducible fragmentation. RNA 6000 Nano kit (Agilent) on a Agilent 2100 Bioanalyzer was used to control for homogeneous fragmentation (50–200 bp range) of samples. Fragmented cDNA was biotinylated at the 3′ termini with GeneChip DNA Labeling Reagent (Affymetrix) and hybridized onto the CauloHI1 chip (Affymetrix). Hybridization was performed at Stanford's Protein and Nucleic Acid facility, as previously described [25]. All data are MIAME compliant and raw data have been deposited in NCBI Gene Expression Omnibus (www.ncbi.nlm.nih.gov/geo/, with series accession number GSE25999).

### Gene annotation

The initial analysis of transcripts and proteins took as a reference the coordinates, ORF prediction and annotation for the *C. crescentus* reference strain CB15 (GeneBank accession AE005673, RefSeq NC_002696). Upon release of the genomic sequence of the *C. crescentus* laboratory strain CB15N (also known as NA1000; GeneBank accession CP001340, RefSeq NC_011916), which includes an updated ORF prediction and gene annotation [75], all results were mapped to this new annotation. Supplementary tables include gene IDs corresponding to both genomes nomenclatures. To generate and process diverse gene lists from the microarrays and proteomics results, a Pathways and Genome Database (PGDB) was built using the PathoLogic and Pathway/Genomes Editor software, in the Pathway Tools platform [76]. The *C. crescentus* NA1000 PGDB was manually curated to incorporate information for diverse published datasets, and is available upon request.

### Statistical analysis of microarray data from carbon starved *C. crescentus* mixed population

The RMA statistical algorithm [77], available under the Bioconductor software package of R, was used for background noise removal, normalization and summarization of microarray data corresponding to two independent experiments for each condition (cells starved for carbon for 30 and 60 min, and the respective controls). All data are MIAME compliant, and raw and normalized data files were submitted to NCBI Gene Expression Omnibus (accession number GSE25996). A Significance Analysis of Microarrays (SAM) [78] was applied to the dataset, with the following parameters: unpaired, logged, median centered, T statistic. A 2-fold minimum change was selected as cutoff, and a delta value yielding a false discovery rate lower than 1%.

### Clustering of transcript changes in swarmer and stalked cells, and search for conserved promoter motifs

Microarray data were normalized and summarized as described in the previous section. All data are MIAME compliant and raw and normalized data has been deposited in NCBI Gene Expression Omnibus (www.ncbi.nlm.nih.gov/geo/, with accession number GSE25997). For each one of the 3767 genes analyzed, we created a profile vector composed of two values: i) log-ratio between expression values measured for swarmer cells after 15 minutes of carbon starvation and the non-starved control, ii) log-ratio between expression values for stalked cells after 15 minutes of carbon starvation and non-starved control. The non-starved controls values were obtained by linearly interpolating the data from [25], at the corresponding time-points (15 minutes into the cell cycle, as a control for swarmer cells, and 75 minutes for stalked cells). We defined a gene as not responding to carbon starvation if the fold-change in expression was less than 2×. Similarly, we defined a gene as responding to carbon starvation if the fold-change in expression was greater than 3×. We only considered genes that, for both carbon starved swarmer and stalked cells, either responded to carbon starvation or did not (no change: −1<|log-ratio|<1; change: |log-ratio|>1.585). We then clustered the profiles of the genes using a bottom-up hierarchical clustering approach (Bioconductor R package), using the Pearson correlation distance and complete linkage for measuring inter-cluster distances. To search for conserved motifs, we extracted 250 bp of sequence (from −200 to +50 with respect to the translational start site) for the genes in each cluster, and used MEME [79], with the following parameters: distribution of motif occurrences: zero or one per sequence; minimum motif width: 6; maximum motif width: 25. We selected the motifs with an E-value< = 0.2 and average Relative Entropy> = 1bit/bp.

### Functional categories enrichment of gene and protein sets

In order to identify functional categories that were significantly over or under-represented within the set of transcripts and proteins that changed upon carbon starvation (30 minutes for transcripts and 30 and/or 60 minutes for proteins), we calculated p-values for each COG-category based on hyper-geometric distributions (p-value< = 1%). Similarly, in order to identify known motifs, associated with cell-cycle and metal stress [25], that were significantly over or under-represented in the upstream regions of the set of genes whose expression changed significantly upon 30 minutes of carbon starvation, we calculated p-values based on hyper-geometric distributions (p-value< = 5%).

### Microarray analysis of Δ*sigT* and Δ*sigU* strains upon starvation

For each microarray experiment, expression signals for 3767 genes were analyzed. The RMA statistical algorithm [77], available under the Bioconductor software package of R, was used for background noise removal, normalization and summarization of the microarray data. All data are MIAME compliant, and raw and normalized data files were submitted to NCBI Gene Expression Omnibus (accession number GSE25998). For each gene i, we calculated the difference between sample means and a two-sample t statistic. For each gene and each condition (Δ*sigT*, Δ*sigU* and wild-type strains starved for glucose for 15 minutes), we then calculated a p-value derived from the gene-specific t-statistic, as well as the empirical distribution derived from the t-statistics of all genes. A gene was considered to be differentially expressed, if two conditions were met: the difference between the mutant and WT was greater than two-fold, and the p-value derived from the corresponding t-statistics was smaller than 5%.

## Supporting Information

Table S1 **Proteome coverage of bacterial species.** The highest proteomic coverages for bacterial organisms are shown with the corresponding reference. The proteomic coverage is expressed in the percentage of annotated genes for which the predicted encoded protein has been detected, as reported by the cited publications.
(DOC)

Table S2 **List of proteins that change significantly upon carbon starvation.** Gene ID (for strains CB15 and CB15N), annotation, COG category (if applicable), fold change and corresponding p-value are shown for the proteins that were found to change reproducibly in cells starved for carbon for 30 and/or 60 min.
(XLS)

Table S3 **List of transcripts that change significantly upon carbon starvation.** Gene ID (for strains CB15 and CB15N), annotation, COG category (if applicable), average fold change and corresponding standard deviation are shown for the transcripts that were found to change reproducibly in cells starved for carbon for 30 and/or 60 min. For each transcript it is also indicated whether it was previously classified as cell cycle regulated [24].
(XLS)

Table S4 **List of genes that change significantly upon carbon starvation in the sets represented in** **Figure 2** **.** Gene ID (for strains CB15 and CB15N), annotation, and fold change between starved and non-starved cells at 30 min (except for motif cc_7, for which the values correspond to 60 min) are shown.
(XLS)

Table S5 **List of genes in the clusters represented in** **Figure 3B** **.** Gene ID (for strains CB15 and CB15N), log-2 ratio of transcript levels of starved and non-starved swarmer (SW) and stalked (ST) cells after 15 min, annotation, and COG category (if applicable), are shown for all genes clustered in Figure 3B.
(XLS)

Table S6 **List of proteins and transcripts that are upregulated upon carbon starvation and heavy metal stress.** Genes that were induced by two or more heavy metals [29], and were upregulated in carbon starvation at the level of protein or transcript.
(XLS)

Table S7 **Lists of genes represented in** **Figure 6** **.** This spreadsheet contains eight separate tabs with the lists of genes corresponding to the categories in the chart in Figure 6. Each list was obtained from the intersections of the lists of genes and proteins determined to be up- and down-regulated according to the criteria described in Methods, for those 1378 genes for which there was valid data at both the transcript and the protein level, and a significant change in at least one of the datasets (microarrays or proteomics).
(XLS)

Table S8 **Lists of genes represented in** **Figure 7** **.** Genes in the FixK carbon starvation regulon were obtained from the intersection of up- and down-regulated genes after 30 min of carbon starvation with genes with motif cc_3 according to McGrath *et. al.* [25], and FixK-dependent genes in hypoxia according to Crosson *et. a*l. [27]. Genes in the LexA carbon starvation regulon were obtained from the intersection of up- (1) and down-regulated (2) genes after 30 min of carbon starvation with genes in the SOS regulon [52]. Genes in the RpoD carbon starvation regulon were obtained from the intersection of up- (1) and down-regulated (2) genes after 30 min of carbon starvation with genes with motif cc_6 according to McGrath *et. al.* [25]. Genes in the SigT carbon starvation regulon are listed in Table 1. The proteins encoded by SigT targets from Table 1 that are upregulated after 30 or 60 min of carbon starvation are listed here. The CtrA regulon was determined by intersecting the list of CtrA targets [24] (Table 2) with the list of genes up- and down-regulated after 30 min of carbon starvation (Table S3), and the list of proteins that are up- or down-regulated after 30 and/or 60 min of carbon starvation (Table S2). Only the genes for which the transcript and protein changed upon starvation (sets 1 and 2) were included in Figure 7.
(XLS)
