# Supplementary material for: Regulatory Response to Carbon Starvation in Caulobacter crescentus
Source: PLoS One. 2011 Apr 11;6(4):e18179. doi: 10.1371/journal.pone.0018179 (PMC3073932; doi:10.1371/journal.pone.0018179)
Supplement: Table S1 — Proteome coverage of bacterial species. The highest proteomic coverages for bacterial organisms are shown with the corresponding reference. The proteomic coverage is expressed in the percentage of annotated genes for which the predicted encoded protein has been detected, as reported by the cited publications. (DOC) [file pone.0018179.s001.doc]

| **Organism** | **Proteome coverage (%)** | **Reference** |
| --- | --- | --- |
| *M. genitalium* | 89 | [1] |
| *C. crescentus* | 66 | This work |
| *L. interrogans* | 64 | [2] |
| *D. radiodurans* | 61 | [3] |
| *Synechocystis sp. PCC 6803* | 53 | [4] |
| *R. sphaeroides* | 39 | [5] |
| *S. aureus* | 32 | [6] |
| *Y. pestis* | 25 | [7] |
| *M. acetivorans* | 24 | [8] |
| *D. vulgaris* | 21 | [9] |
| *M. capsulatus* | 18 | [10] |
| *S. oneidensis* | 14 | [11] |
| *S. enterica* | 7 | [12] |

**Table S1. Proteome coverage in bacterial species.**

1. Jaffe JD, Stange-Thomann N, Smith C, DeCaprio D, Fisher S, et al. (2004) The complete genome and proteome of Mycoplasma mobile. Genome Res 14: 1447-1461.

2. Cao XJ, Dai J, Xu H, Nie S, Chang X, et al. (2010) High-coverage proteome analysis reveals the first insight of protein modification systems in the pathogenic spirochete Leptospira interrogans. Cell Res 20: 197-210.

3. Lipton MS, Pasa-Tolic L, Anderson GA, Anderson DJ, Auberry DL, et al. (2002) Global analysis of the Deinococcus radiodurans proteome by using accurate mass tags. Proc Natl Acad Sci U S A 99: 11049-11054.

4. Wegener KM, Singh AK, Jacobs JM, Elvitigala TR, Welsh EA, et al. (2010) Global proteomics reveal an atypical strategy for carbon/nitrogen assimilation by a cyanobacterium under diverse environmental perturbations. Mol Cell Proteomics.

5. Callister SJ, Nicora CD, Zeng X, Roh JH, Dominguez MA, et al. (2006) Comparison of aerobic and photosynthetic Rhodobacter sphaeroides 2.4.1 proteomes. J Microbiol Methods 67: 424-436.

6. Scherl A, Francois P, Charbonnier Y, Deshusses JM, Koessler T, et al. (2006) Exploring glycopeptide-resistance in Staphylococcus aureus: a combined proteomics and transcriptomics approach for the identification of resistance-related markers. BMC Genomics 7: 296.

7. Hixson KK, Adkins JN, Baker SE, Moore RJ, Chromy BA, et al. (2006) Biomarker candidate identification in Yersinia pestis using organism-wide semiquantitative proteomics. J Proteome Res 5: 3008-3017.

8. Li L, Li Q, Rohlin L, Kim U, Salmon K, et al. (2007) Quantitative proteomic and microarray analysis of the archaeon Methanosarcina acetivorans grown with acetate versus methanol. J Proteome Res 6: 759-771.

9. Nie L, Wu G, Zhang W (2006) Correlation between mRNA and protein abundance in Desulfovibrio vulgaris: a multiple regression to identify sources of variations. Biochem Biophys Res Commun 339: 603-610.

10. Kao WC, Chen YR, Yi EC, Lee H, Tian Q, et al. (2004) Quantitative proteomic analysis of metabolic regulation by copper ions in Methylococcus capsulatus (Bath). J Biol Chem 279: 51554-51560.

11. Fang R, Elias DA, Monroe ME, Shen Y, McIntosh M, et al. (2006) Differential label-free quantitative proteomic analysis of Shewanella oneidensis cultured under aerobic and suboxic conditions by accurate mass and time tag approach. Mol Cell Proteomics 5: 714-725.

12. Shi L, Adkins JN, Coleman JR, Schepmoes AA, Dohnkova A, et al. (2006) Proteomic analysis of Salmonella enterica serovar typhimurium isolated from RAW 264.7 macrophages: identification of a novel protein that contributes to the replication of serovar typhimurium inside macrophages. J Biol Chem 281: 29131-29140.
